# A Review on Natural Antioxidants for Their Role in the Treatment of Parkinson’s Disease

**DOI:** 10.3390/ph16070908

**Published:** 2023-06-21

**Authors:** Pooja Mittal, Sanchit Dhankhar, Samrat Chauhan, Nitika Garg, Tanima Bhattacharya, Maksood Ali, Anis Ahmad Chaudhary, Hassan Ahmad Rudayni, Mohammed Al-Zharani, Wasim Ahmad, Salah Ud-Din Khan, Thakur Gurjeet Singh, Somdutt Mujwar

**Affiliations:** 1 Chitkara College of Pharmacy, Chitkara University, Rajpura 140401, India; poojamittal2009@gmail.com (P.M.); sanchitdhankhar@gmail.com (S.D.); samrat.chauhan11@gmail.com (S.C.); gurjeet.singh@chitkara.edu.in (T.G.S.); somduttmujwar@gmail.com (S.M.); 2 Ganpati Institute of Pharmacy, Bilaspur 135102, India; 3 Department of Food and Nutrition, BioNanocomposite Research Center, Kyung Hee University, 260 Kyunghee-daero, Seoul 02447, Republic of Korea; 4 Nondestructive Bio-Sensing Laboratory, Department of Biosystems Machinery Engineering, College of Agriculture and Life Science, Chungnam National University, 99 Daehak-ro, BLDG# E10-2, RM# 2213, Daejeon 34134, Republic of Korea; 5 Department of Pharmacognosy, Orlean College of Pharmacy, Dr. A.P.J. Abdul Kalam Technical University, 42, Knowledge Park—III, Greater Noida 201308, India; drmali_pharmacy13@yahoo.com; 6 Department of Pharmacognosy, HIMT College of Pharmacy, Dr. A.P.J. Abdul Kalam Technical University, 8, Institutional Area, Knowledge Park—I, Greater Noida 201301, India; 7 Department of Biology, College of Science, Imam Mohammad Ibn Saud Islamic University (IMSIU), Riyadh 11623, Saudi Arabia; harudayni@imamu.edu.sa (H.A.R.); mmyzharani@imamu.edu.sa (M.A.-Z.); 8 Department of Pharmacy, Mohammed Al-Mana College for Medical Sciences, Dammam 34222, Saudi Arabia; wasimahmadansari@yahoo.com; 9 Department of Biochemistry, College of Medicine, Imam Mohammad Ibn Saud Islamic University (IMSIU), Riyadh 11623, Saudi Arabia; sdikhan@imamu.edu.sa

**Keywords:** neurodegeneration, antioxidants, phytoconstituents, polyphenols, Parkinson’s disease

## Abstract

The neurodegenerative condition known as Parkinson’s disease (PD) is brought on by the depletion of dopaminergic neurons in the basal ganglia, which is the brain region that controls body movement. PD occurs due to many factors, from which one of the acknowledged effects of oxidative stress is pathogenic pathways that play a role in the development of Parkinson’s disease. Antioxidants, including flavonoids, vitamins E and C, and polyphenolic substances, help to reduce the oxidative stress brought on by free radicals. Consequently, this lowers the risk of neurodegenerative disorders in the long term. Although there is currently no cure for neurodegenerative illnesses, these conditions can be controlled. The treatment of this disease lessens its symptoms, which helps to preserve the patient’s quality of life. Therefore, the use of naturally occurring antioxidants, such as polyphenols, which may be obtained through food or nutritional supplements and have a variety of positive effects, has emerged as an appealing alternative management strategy. This article will examine the extent of knowledge about antioxidants in the treatment of neurodegenerative illnesses, as well as future directions for research. Additionally, an evaluation of the value of antioxidants as neuroprotective agents will be provided.

## 1. Introduction

**Figure 1:**
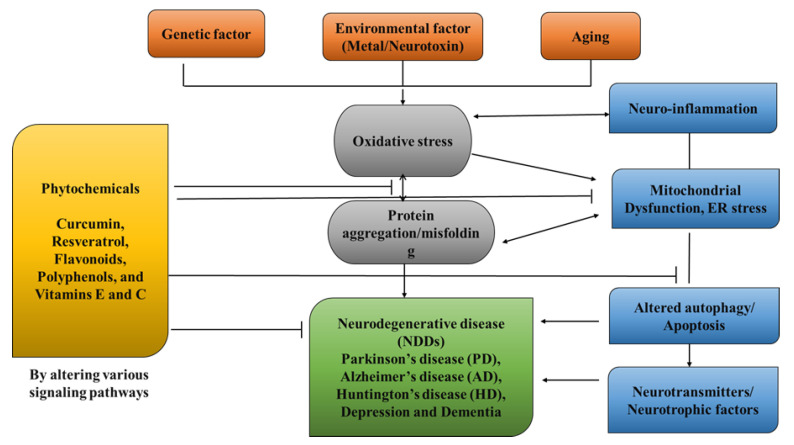
Role of Phytochemicals in the treatment of neurological diseases.

NDDs (neurodegenerative disorders) are a collection of disorders with various clinical implications and etiology. ALS (amyotrophic lateral sclerosis), cerebellar illness, Huntington’s chorea, Parkinson’s disease, Alzheimer’s, dementia, and schizophrenia are all examples of NDDs [1,2,3,4]. Age groups, hereditary diseases, non-enzymatic antioxidants, excitotoxicity, cytoskeletal abnormalities, autoimmunity, asymmetry, toxicity, elevated blood pressure, oxidative stress, and peripheral vascular diseases are subject to both experimental and epidemiological research. Some of the risk variables that have been discovered through the clinical manifestations of NDDs are associated with free radical toxicity, radical-mediated alteration, oxidase dysfunction, and endoplasmic reticulum stress caused by perinatal genetic abnormalities. The most important common symptoms are disturbances in balance, breathing, movement, reflexes, motor skills, or cardiac activity. Antioxidants such as flavonoids, polyphenols, and vitamins E and C can help to prevent these symptoms [5,6,7]. Antioxidants have a great impact on human health, as they can fight free radicals and, thus, halt the aging process and decrease the effects of oxidative damage caused by, for example, an unbalanced diet [8,9]. As a result, the long-term risk of neurodegenerative diseases is reduced (Figure 1). Although they can be treated, there is currently no cure for neurodegenerative diseases. Treatment of this disease relieves the symptoms in order to preserve the quality of life. Natural antioxidants, such as polyphenols, are becoming more popular because they can be obtained from foods and supplements and offer a range of health benefits [10]. Although it affects both males and females with increase in their age but males tend to be affected more frequently [11]. It is considered an international disease that does not distinguish between social class or race. The disease is estimated to affect 1% of people over the age of 65 worldwide, accounting for up to two-thirds of all people with movement disorders [12].

As the population ages, PD has become more prevalent, with those over 85 years old reaching 2.6%. The non-motor symptoms that patients with PD encounter include pain, exhaustion, autonomic dysfunction, changed mood, sleep disturbance, and cognitive abnormalities [13]. PD is referred to asa synucleinopathy because Lewy bodies, a crucial clinical characteristic of the disease, accumulate due to the misfolding of β-synuclein as a major feature. Furthermore, β-synuclein appears to be connected to both idiopathic and inherited forms of Parkinson’s disease and has a special role in the disease’s pathogenesis. It is worth noting that β-synuclein buildup has been closely linked to posttranslational modifications, systemic inflammation, oxidative stress, mitochondrial biogenesis, changed mitochondrial physical properties, synapse dysfunction, glycolipids, ER stress, and metal complexes. The overproduction of ROS and years of age breakdown the antioxidant defense system and increase oxidative stress in certainbrain areas, which can contribute to the misfolding of β-synuclein being the catalyst for the aging process in PD [14,15]. Although it is not a cure, levodopa has emerged as an efficacious drug for PD’s first motor symptoms. Despite the fact that stiffness and bradykinesia respond the best, tremors may only be somewhat reduced by levodopa. Other symptoms, such as balance issues, may worsen. L-dopa, however, cannot be used to treat Lewy disease, non-motor symptoms, or neuronal loss [16]. Patients require greater L-dopa doses over time, which is accompanied by a rise in side effects such as dyskinesias. Amantadine, an antiviral medication, appears to alleviate motor problems as well. Medicines based on dopamine agonists are also administered to address a range of neuromotor symptoms that are associated with disease progression. The long-term use of conventional PD medications can lead to adverse effects, such as dyskinesias and motor fluctuations (Table 1). As a result, novel therapeutic techniques used to prevent neurodegeneration, non-motor symptoms, Lewy disease accumulation, or synuclein aggregation in the brain are required [17,18].

**Complementary Therapies for PD**

**Figure 2:**
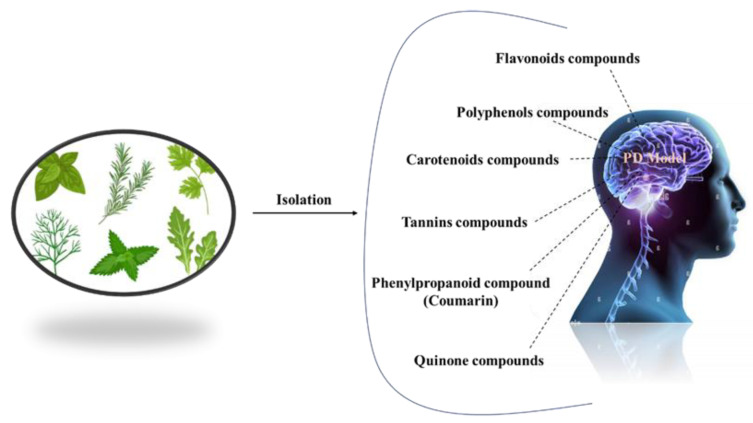
Different natural antioxidants used in the treatment of PD.

Complementary therapies and phytonutrients derived from plant sources have been proposed as treatments for Parkinson’s disease [24]. Numerous natural phytochemicals have emerged as therapeutically interesting compounds, drug entities, and phytochemicals for the treatment of inflammatory disorders [25]. Additionally, numerous pharmacological studies have shown that phytochemicals are useful in treating neurodegenerative diseases (NDDs), depression, and dementia [26,27,28,29,30]. Physiologically active phytochemicals are important therapeutically because they serve as the antioxidant defense system’s primary and secondary metabolites, which protect against a variety of stress-related disorders and clinical symptoms [31]. These phytochemicals’ positive and therapeutic effects include antioxidant capacity, pathogen prevention, immune system activation, and nutritional support for healthy living cells [32]. Botanical substances, with their active phytonutrients, efficiently salvage oxygen-based free radicals, influencing the antioxidative defense system and contributing to major cognitive impairments such as dyskinesias, residual tremors, muscle stiffness, and instability [33]. This review holds prime importance, as it addresses the treatment gaps in PD and explores the potential of natural antioxidants as a therapeutic approach. By summarizing the current literature on natural antioxidants and their effects on PD, this review aims to provide a comprehensive overview that can guide further research and clinical trials. Understanding the role of natural antioxidants in PD treatment may lead to the development of novel therapeutic strategies that can modify the disease course, mitigate the symptoms, and improve the quality of life for individuals living with PD. In this review, we address the natural antioxidants that can be used to treat Parkinson’s disease, as well as their mode of action (Figure 2).

**Pathogenesis**

**Figure 3:**
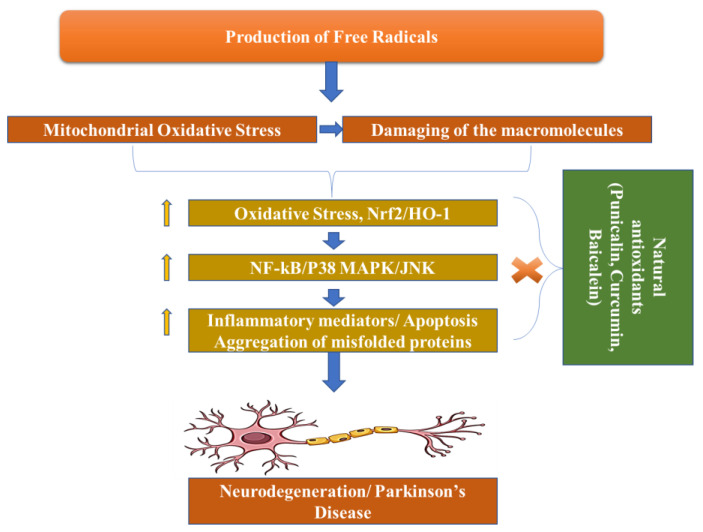
Pathogenesis of Parkinson’s Disease.

Dopamine levels in the caudal nucleus, striatum, and expression medium fall as a result of the death of dopaminergic neurons in the substantia nigra pars compacta, which is associated with Parkinson’s disease. The progressive loss of cholinergic neurons is the most significant pathogenic discovery in Parkinson’s disease sufferers’ brains. For this reason, with the loss of these neurons, the dopamine levels fall. When 50–60% of the dopamine pathway is shut down and striatal dopamine levels are lowered by 80–85%, symptoms of the disease arise. However, Parkinson’s disease may have multiple etiological factors, including mitochondrial dysfunction, neuroinflammation, oxidative stress, and the formation of protein kinases called Lewy bodies in the cytosol (Figure 3) [34].

**Phytochemicals and Parkinson’s Disease**

Neurodegenerative diseases, including Parkinson’s disease, contributes to N increase in nicotinamide adenine dinucleotide phosphate oxidase and intracellular RNS, and, thus, result in cell damage due to oxidative stress [35].

Free radicals also damage cell membranes by oxidizing proteins, lipids, and nucleic acids, causing peptide cross-links [36]. The antioxidant and chelating effects of flavonoids and terpenoids are believed to be accountable for their therapeutic effects. Terpenoids, phenols, and flavonoids show important neuroprotective effects, due to their oxidative capacity [37]. By releasing extra protons, flavonoids scavenge superoxide, hydroxyl, and peroxyl radicals by donating an extra proton. This prevents ROS generation by the formation of complex structures containing dihydroxy groups, copper, multiple ions of transition metals, and iron [38].

Glutathione reductase, septo-optic dysplasia, and glutathione-S-transferase (GST) are the polyphenols that activate antioxidant enzymes. Polyphenols can raise the antioxidant levels by enhancing cell-signaling pathways through these enzymes. By generating unsaturated carbon–carbon double bonds with lipid bilayers, these phenolic acids inhibit lipid peroxidation and, thus, work as a powerful therapeutic and preventative agent [39].

The lack of oxygenation of the rings is assumed to be responsible for chrysin’s chemical capabilities, which include anti-inflammatory and antioxidant potential [40]. Diverse structures of flavones have been discovered to increase the capacity of antioxidant enzymes and also work in inhibiting COX-2, which is a pro-inflammatory mediator. Experiments with xanthine oxidase demonstrate that chrysin dramatically reduces ROS production. It is worth noting that rodent models require polyphenol dosages ranging from 10 µM to 100 µM to exercise their putative antioxidant and anti-inflammatory properties [41].

**Neuroprotective Dietary Antioxidants**

**Figure 4:**
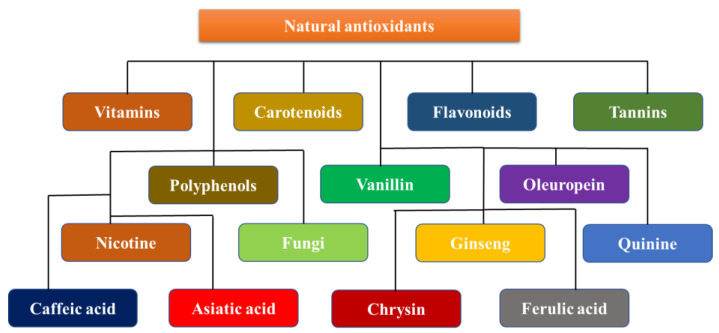
Neuroprotective dietary antioxidants.

Antioxidants are neuroprotective compounds that protect or repair cellular components from oxidative damage. They efficiently halt neuronal lipid, protein, and DNA breakdown. Vitamins, flavonoids, carotenoids, tannins, and polyphenols are all antioxidants [42]. Oxidative stress damage is a prominent manifestation of neurodegenerative disorders. However, few efficacious agents have been found for clinical use, and even fewer have been successful because of their toxicity and cancer risks [43]. Evidence suggests that neuroprotection is a potential pharmaceutical target for neurodegenerative diseases, suggesting that it is possible. The use of relatively harmless antioxidant molecules found in food as remedies for many ailments, on the other hand, is appealing; however, it is limited by the difficulty of creating positive feedback in the brain. Polyphenols, a class of antioxidants, have been shown to have neuroprotective properties. Many biological processes, such as interactions with metal complexes, the scavenging of free radicals, changes in the activity of different enzymes, and impacts on intracellular signaling pathways and gene expression are all biological processes that contribute to this protection. A diet that is high in antioxidants is essential to prevent many diseases [44]. A reduction in the incidence of stroke, neurological disease, heart disease, cancer, and high blood pressure has been associated with these chemicals, which are primarily present in vegetables and fruit. The body’s natural antioxidant system needs help from the exogenous antioxidants that are found in fruits, nuts, vegetables, and other foods. Exogenous antioxidants are often classified as vitamins and non-vitamins. This section reviews the specific properties and roles of the key exogenous antioxidants in neuroprotection (Figure 4) [45].

**Vitamin E**

Four tocopherols, trocochromanols or four tocotrienols, and major fat-soluble antioxidants are collectively referred to as vitamin E [46]. The human body cannot produce this vitamin itself, therefore, it must be obtained from food (Table 2).

Each vitamin E isoform has complex antioxidant properties, and it is currently unknown how these effects work. It is suspected that they work by stopping the chain process of lipid peroxidation, therefore, important cellular components can be protected [47]. Vitamin E bioavailability varies with many factors, including the dietary matrix, genetics, and metabolic fat, and ranges from 10 to 33%. Preclinical studies in Parkinson’s disease have shown conflicting results regarding the efficacy of vitamin E as a disease-modifying agent [48]. Vitamin E deficiency increased substantia nigra MPTP toxicity, while partially protecting against neurotransmitter and metabolite deficiencies caused by striatal MPTP. Dopamine degradation could not be stopped, even by pre-MPTP injection therapy with β-tocopherol. However, substantial amounts of vitamin E may partially protect dopaminergic neurons from MPTP-mediated toxicity [37]. During the Deprenyl and Tocopherol antioxidative treatment of Parkinsonism study, patients with early or untreated Parkinson’s disease were given deprenyl (10 micrograms daily) and tocopherol as a treatment. The results of the study demonstrated that the monoamine oxidase inhibitor (deprenyl) slowed the progression of the disorders.However, the modest improvement in motor function gradually diminished when deprenyl was discontinued [49]. In individuals with Parkinson’s disease with a recent diagnosis, vitamin C and alpha-tocopherol supplementation delayed the initiation of levodopa therapy by 25 years.

**Table 2:** Some other potent activities of Vitamin E.

Compound Name	Disease	Impact	Ref.
Vitamin E	Alzheimer’s disease (AD)	Effective against AD when consumed with vitamin C in the earlier phase of AD.	[50]
	Cell membrane dysfunction	Effective in the protection of the cell membrane	[51]
	Cardiovascular disorder	Prevents the clot formation and buildup of bad cholesterol	[52]

**Ascorbic Acid**

Ascorbate, or ascorbic acid, is a vitamin that is soluble in water that is necessary for catecholamine production and other enzyme processes. Ascorbic acid shows its antioxidant capabilities by reduction inO2, alkoxyl (RO), hydroxyl (HO), peroxyl (ROO), and other free radicals in body. When vitamin E is oxidized while scavenging free radicals from the lipid membranes, the radical tocopheroxyl is formed, which interacts with vitamin C in order to replenish vitamin E. Vitamin C absorption, distribution, and metabolism are more complex than those of most lighter weight molecular compounds [53]. A family of sodium-dependent vitamin C transporters contains the key proteins that are involved in substance distribution and tissue absorption. The brain contains one of the highest concentrations of vitamin C in the body. Concentrations vary per brain region, with the motor cortex having the lowest concentration [54]. Different transporters are present in the brain for reduced and oxidized forms of vitamin C (SVCT2 or GLUT family). Individuals whosuffer from neurodegenerative conditions such as Parkinson’s disease (PD) exhibit low plasma exogenous antioxidant concentrations (including vitamin C). A lack of vitamin C has also been related to a higher incidence of Parkinson’s disease. It has been proposed that the level of vitamin C in lymphocytes could serve as a diagnostic indicator of Parkinson’s disease progression. Vitamin C supplementation is one of the medicinal methods used to cure Parkinson’s disease [55] (Table 3).

**Flavonoids**

It is the largest polyphenol class and has a backbone that is made up of one heterocyclic ring and two phenyl rings. The side chain configuration and quantity of hydroxyl groups influence the biological features, such as antioxidant activity and the modification of enzyme activity [40].

By reacting with free radicals and serving as metal catalysts, flavonoids act as radical chain terminators. In order to inhibit oxidation, phenolic antioxidants (PhOH) provide hydrogen atoms to the free radicals. There are several different types of flavonoids present in legumes, plants, and fruits (obtained from celery, citrus peels, chamomile, paprika, ginkgo biloba, parsley leaves, and mint). Crops, flowers, and leaves contain flavones as glucosides [59].

Kaempferol, quercetin, ferulic acid, and myricetin are examples of flavonols. In 6-OHDA-treated PC12 cells, 20 µM of quercetin boosted the mitochondrial activity, decreased the oxidative stress, and decreased β-synuclein protein synthesis [60]. Furthermore, oral quercetin administration improved the motor performance in arat model by lowering the mitochondrial oxidative stress, β-synuclein aggregation formation, and neuronal cell death [46].

Chalcone, isoflavonoids (such as daidzein and genistein), water-soluble anthocyanidin pigments (such as delphinidin, pelargonidin, peonidin, and cyanidin), and aromatic colorless flavanones (such as hesperetin, naringin, and hesperidin) are the other class of flavonoids that can be useful in the treatment of PD (Table 4) [61].

**Green Tea Polyphenols**

Green tea polyphenols, notably EGCG, exhibit a broad range of pharmacological properties, involving antimutagenic and anticarcinogenic properties. Green tea polyphenols’ strong antioxidant capacity is responsible for these positive effects. They are potent antioxidants that combat free radicals such as lipid radicals, phenolic radicals, superoxide anion radicals, and hydroxide ions [65]. When they are taken orally, the flavonoids and green tea’s polyphenols have been demonstrated to prevent aging. Tea catechins (TC) are commonly thought to be radical scavengers, although other components of tea have numerous applications, possibly protecting against 6-OHDP-induced apoptosis. In MTT assays, 6-OHDA treatment decreased the cell viability in a concentration-dependent manner, whereas flow cytometry, fluorescence microscopy, and DNA fragmentation confirmed apoptosis in PC12 cells. TC substantially reduces PC12 cell death. EGC and EC had lower efficacy, but EGCG and ECG were more efficacious compared to TC. The protective effect increased from 50 µM to 400 µM, and, at these concentrations, EGCG outperformed the green tea polyphenols. They inhibit at 83.2% and 84.9%, respectively, while EGCG inhibitory concentration is at 88.4% and 90.2% [54]. According to flow cytometry data, 200–400 µM of green tea polyphenols and EGCG dramatically decrease the number of apoptotic cells. Notably, EGCG-protected PC12 cells abolished nuclear changes indicative of apoptosis [66] (Table 5).

**Isoflavones**

Genistein, the most active soy isoflavone, has estrogen receptor affinity, antioxidant characteristics, increased cytoplasmic glutathione peroxidase, PTK inhibition, and other physiological effects. Soy isoflavones have protective efficacy against a variety of illnesses, including atherosclerosis, the consequences of estrogen deficiency in menopause, and hormone-dependent breast and prostate cancer (Table 6) [70]. It has been found that it exhibits neuroprotection by antagonizing the toxic effect of the critical Aβ protein [71]. As a result, it can be used for PD management.

**Nicotine**

In hippocampus cultures, nicotine significantly inhibits apoptosis induced by Ab and increases caspase activity. Nicotine reduced the number of free radicals accumulated by the Ab. The cholinergic antagonist mecamylamine decreased nicotine’s capacity to protect cells from Ab-induced caspase-3 activation and ROS buildup [75]. According to research, nicotine receptors play a major role in nicotine’s protective potential. The findings imply that nicotine may assist in averting the emergence of neurodegenerative diseases such as PD and Alzheimer’s. Although smoking has been linked to a lower incidence of Parkinson’s disease, the specific mechanism for this is unknown. Nicotine has been discovered to suppress cytochrome C release from intact mitochondria, as well as the high-amplitude mitochondrial swelling that is caused by calcium and *N*-methyl-4-phenylpyridine (MPPt). The redox state within mitochondria was also maintained by nicotine, which was likelyas a result of decreased mitochondrial permeability. Nicotine did not prevent the reduction in mitochondrial membrane potential by MPPt or calcium, but decreased electron leakage at sites of respiratory chain complex I and 6-OHDA, causing cytochrome C release and mitochondrial enlargement in SH-SY5Y cells [66].

This suggests that nicotine has receptor-independent neuroprotective effects. These findings imply that, when evaluating nicotine’s neuroprotective benefits, it is critical to consider both its interaction with the mitochondrial respiratory chain and its antioxidant activity. It is not entirely clear how nicotine attenuates β-amyloidosis and prevents PD.

In one study, after nicotine treatment, pure zincand copper metal concentrations in amyloid plaques and neuropils dropped considerably. The density of zinc and copper distribution in the hippocampus CA1 area is likewise lowered after nicotine administration. Researchers examined how nicotine affected the homeostasis of metals in SH-SY5Y cells that were overexpressing the Swedish variant of human APP (APPsw). These effects are not dependent on nicotinic acetylcholine receptor activation and are mediated by a decrease in intracellular copper concentrations with nicotine therapy, as well as a reduction in Ab-induced neurotoxicity, helped by copper addition [76] (Table 7).

**Carotenoids**

There are about 850 fat-soluble tetraterpenes in the class of carotenoids that are produced by photosynthetic plants, algae, and bacteria. They support plants in both photosynthesis and photo-defense. In addition to fruits and vegetables, such as beets, cherries, melons, and pumpkins, foods such as microalgae, fish, and shrimp also contain yellow, orange, and red pigments called carotenoids [80]. Serum lycopene, β, and α-carotene levels are considerably lower in Parkinson’s disease patients, and lower serum carotenoid levels are also linked to motor impairment. Carotenoids act as antioxidants by eliminating ROS via chemical and physical reactions; for example, β-carotene absorbs thermal energy from singlet oxygen and/or peroxynitrite, therefore, inhibiting tyrosine nitration. Carotenoids protect neurons against neurodegenerative diseases in multiple ways, including ROS quenching, the activation of antioxidant enzyme systems, and anti-neuroinflammatory effects [81] (Table 8).

**Resveratrol**

Resveratrol is a phenolic compound chemical that is accessible in several plants, including berries and grapes. In animal studies of Parkinson’s disease, they help with movement deficits, oxidative stress, and the elimination of TH neurons [85]. Resveratrol inhibits chromatin condensation and mitochondrial expansion, while decreasing COX-2 and TNF gene output [86]. This phenol has a short oral survival period due to its low water solubility and short half-life (10 min). Furthermore, resveratrol derivatives triethylsilyl and ditriisopropylsilyl show superior action in invitro anti-inflammatory and neuroprotective properties when compared to pure resveratrol. Resveratrol is employed because it has shown increased biocompatibility and neuroprotective properties in zebrafish embryos. Also selected from the prodrugs, this chemical reduced clinical scores and the severity of motor impairment in a 3-nitropropionic acid PD mousemodel of experimental autoimmune encephalomyelitis [87] (Table 9).

**Tannins**

These phenolic compounds have enough hydroxyl and carboxyl groups and high molecular weight to form a complex with biomolecules (0.5–20 kDa). They are also water-soluble. Tannins are unique in their ability to precipitate and link to alkaloids, proteins, and amino acids.There are several methods for removing tannins, however, acetone solvent extraction is the most effective for reducing tannin–protein complexes [91]. Tannic acid was chosen as the most optimistic molecule to minimize α-synuclein fibrosis without significantly raising toxicity levels [92] (Table 10).

**Curcumin Polyphenol**

Curcumin (10 µM) therapy also lowers oxidation-related protein changes such as carbonylation and nitrotyrosine production, which help to sustain dopaminergic cells. Curcumin, an antioxidant, effectively protects against oxidative-stress-induced mitochondrial damage [96,97]. In MPTP-induced mouse striatum, curcumin inhibits TH-positive cell death and DA depletion. In addition, it reduces inflammatory markers such as total nitrite, cytokines, and inducible nitric oxide synthase [98] (Table 11).

Herbs’ most prevalent chemical ingredients are ginkgolic acids, terpenoids, and flavonoids. Quercetin, isorhamnetin, and kaempferol are the most prevalent flavonoids in a registered standardized extract of ginkgo biloba (EGb 761) produced by solid–liquid extraction using aqueous acetone. Terpene lactones account for 6% of the extract (3.1% ginkgolides and 2.9% bilobalides) and other components (e.g., glucose and organic acids).

The long-term injection of EGb761 reduced dopaminergic nerve terminal degeneration caused by 1-methyl-4-phenyl-1,2,3,6-tetrahydropyridine in a Parkinson’s disease rat model (MPTP) [102]. Before and after treatment, EGb761 has been shown to guard against MPTP-induced dopaminergic neurotoxicity. Furthermore, EGb761 reduced the neurotoxicity of levodopa in the Parkinson’s disease 6-hydroxydopamine (6-OHDA) model (PD). Monoamine oxidase, an enzyme that is involved in dopamine metabolism and the production of free radicals that damage nigrostriatal neurons, was either inhibited or lowered by EGb761 [103] (Table 12).

**Pomegranate**

Punica granatum, belonging to the Punica family, and the earliest known edible fruit, is endemic to Iran. It is high in polyphenolic chemicals. Pomegranate juice contains soluble polyphenolic components such as catechins, anthocyanins, ellagitannins, gallic acid, and ellagic acid. The anti-inflammatory, antiapoptotic, and antioxidant efficacy of the juice are supported by ellagic acid, punicalin, punicalagin, pedunculagin, gallic acid, glucose esters of ellagic acid, and their metabolites. Ellagitannins are primarily responsible for the neuroprotective properties [107]. In a dose-dependent way, the oral pre-administration of pomegranate extract standardized to 40% ellagic acid shields adult rats against the neuronal damage that is brought on by cerebral ischemia-reperfusion brain injury. The neuroprotective antiapoptotic effect of the extract was in addition to oxidative stress-induced apoptosis, reduce levels of TNF and caspase-3-attributed nuclear factor NF-B p65, and increased interleukin-10 and brain ATP [108] (Table 13).

**Baicalein**

A substance called baicalein (Lamiaceae) is created from the dehydrated roots of *Scutellaria baicalensis*. When it was tested for rotenone-induced neurotoxicity, baicalein decreased ROS generation, apoptosis, ATP deficit, and mitochondrial transmembrane breach PC12 cells [112]. Baicalein treatment raises and maintains dopamine and 5-hydroxytryptamine levels in the basal ganglia. In Hela and SH-SY5Y cells, baicalein reduced α-synuclein oligomerization and aggregation [113] (Table 14).

***Peganum harmara***

*Peganum harmara* (Nitralaceae) reduced muscle stiffness, prevented the oxidation of brain proteins and lipids, and stopped the deterioration of dopaminergic neurons. The herb’s ability to reduce angiotensin II activity is thought to provide neuroprotective effects. The opaminergic neurons are protected and oxidative damage is reduced [116] (Table 15).

***Carthamus tinctorius* L. (Safflower)**

Safflower (Asteraceae), which contains flavonoids, is widely used in China as a traditional remedy for diseases of the cerebrovascular system [112]. In addition to increasing DA levels, DJ-1 and DA transporter expression were also improved. Safflower can reduce the reactive astrogliosis, overexpression, or aggregation of β-synuclein [120] (Table 16).

***Pueraria lobata***

Puerarin (legume family) has been found to inhibit ubiquitin-binding protein accumulation, proteasome malfunction, and the creation of other potentially hazardous proteins. Contrarily, puerarin lowers the ratio of caspase-3 activity to that of bcl-2/bax [124]. Puerarin, is a drug that protects DA, and also safeguards tyrosine hydroxylase (TH)-positive neurons from the damage that is caused by 6-OHDA [125] (Table 17).

**Ginseng**

Rb1 and Rg1 ginsenosides are most likely the primary ginseng mediators (Araliaceae). In SNK-SH cells, MPTP-induced cell death was prevented by ginsenosides Rb1 and Rg1 (neuroblastoma cell line). By the upregulation of Bcl-2 and Bcl-xl, the downregulation of Bax and iNOS, and the inhibition of caspase-3 activation, Rg1 protects the cells from MPTP-induced apoptosis. Ginsenosides protect cells by increasing the antioxidant activity, lowering intracellular reactive oxygen species (ROS), maintaining complex I activity, and increasing intracellular adenosine triphosphate (ATP) [129].

In mice that were given MPTP, Rg1 enhanced their movement and boosted dopaminergic neurons in the striatum and substantia nigra (SN). Increased ginsenoside Rb1 levels can also prevent α-synuclein polymerization and disintegrate fibrils [130] (Table 18).

***Passionflower***

*Passionflower*, or *Passiflora incarnata*, contains glycosides, flavonoids, alkaloids, and phenolic chemicals. It has the potential to treat a variety of conditions such as anxiety, epilepsy, restlessness, and muscle spasms [134]. Tacrine, a well-known animal model for Parkinson’s tremors, induces jaw movements that can be reduced by passionflower extract. The duration of haloperidol-induced catalepsy in animals was significantly shortened, and cognitive performance improved. The antioxidant activity of this herb is responsible for its PD effects [135] (Table 19).

**St. John’s Wort**

The active ingredients in this plant include naphthodianthrone, phloroglucinol, flavonoids, and essential oils. St. John’s wort (Hypericaceae) also has antioxidant and neuroprotective properties [138]. St. John’s wort extract decreased the neurotoxicity that was produced by long-term rotenone therapy in rats. St. John’s wort reduces the damage caused to neurons and also prevents apoptosis by lowering the Bax levels. After dopaminergic neuron lesioning in rats, it lowered striatal malondialdehyde levels, catalase activity, glutathione (GSH) content, tumor necrosis factor-alpha (TNF-) expression, and DNA fragmentation [139] (Table 20).

***Bacopa monnieri***

The efficacy of *B. monnieri* against Parkinson’s disease was studied in vitro, as well as in animal models of the neurodegenerative disease. *B. monnieri*’s antioxidant and neuroprotective characteristics result in anti-parkinsonian effects linked toreduced synuclein protein aggregation and selective dopaminergic neurodegeneration. In Ayurvedic medicine, this herb is also often used as a brain stimulant. Because of its antioxidant capacity and restoration of mitochondrial ETC complex activity, *B. monnieri* extract has been demonstrated to greatly diminish paraquat-induced Parkinson’s disease in both Drosophila and mice models [143] (Table 21).

***Mushrooms***

Metabolites from sea *Mushroom*s may have anti-PD neuroprotective effects. Neoechinulin A is an isoprene quinone alkaloid that can be isolated from two Aspergillus sp. red algal-based fungi, as well as Microsporum [148]. By addressing mitochondrial complex I malfunction, it can protect PC12 cells from neuronal cell death caused by MPP+ and peroxynitrite [149].

Secalonic acid A is a natural substance derived from two species of marine fungi, Aspergillus ochraceous and Paecilomyces [150]. By blocking p38 phosphorylation, JNK, calcium entry, and caspase-3 activation in colchicine-induced mortality of cortical neurons were significantly reduced at concentrations of 3–10 µM [151] (Table 22).

***Sea Cucumbers***

Nutrient-dense foods include *sea cucumbers* and sea mollusks. In many oriental countries, *sea cucumbers* are considered a restorative and conventional therapy for neurological diseases. These compounds dramatically reduce the 6-OHDA-induced loss of DA neurons in Caenorhabditis elegans [155]. These extracts may also increase lipid levels and inhibit synuclein aggregation. *Cucumaria frondosa* sea cucumber extract has high levels of sphingolipids SCG-1, SCG-2, and SCG-3, which raises TrkA phosphorylation and BDNF expression [156], thus, promoting their potent anti-PD action (Table 23).

**Oleuropein**

Oleuropein is made up of the following three components: elenolic acid, glucose, and hydroxytyrosol. It is a well-known phenolic component that is present in olive oil [160]. Although oleuropein is the most abundant, olive oil also contains aglycones and oleurosides, as well as other oleuropein derivatives, such as beta-6-sulfonic acid, and a radical scavenger of 2,2-diphenyl-1-picrylhydrazyl radicals. The use of oleuropein and its derivatives has been found to minimize ROS accumulation [161,162] (Table 24).

**Theaflavin**

Polyphenol theaflavin (TF) improves the flavor and color of black tea [166]. Because of its antioxidant capabilities, which include the capacity to minimize excessive free radical formation and metal chelation, it is widely known for having a wide range of positive effects against many disorders [167]. According to recently released scholarly research, it may have neuroprotective properties. Theaflavin has been shown to be similarly as efficient as EGCG in reducingamyloid- and α-synuclein-induced neurotoxicity because of its potential antioxidant properties [168] (Table 25).

**Caffeic Acid**

Caffeic acid (CA), a phenol molecule that is present in a variety of vegetation types, is prevalent in caffeine and wines, as well as brews. It contains therapeutic qualities that are similar to propolis, whose remarkable antioxidant potential has been well studied [173]. It also contains pharmacological potential properties, such as anti-cancer, anti-inflammatory, and neuroprotective qualities [174]. It has been discovered that it can interact with the peroxy radicals that are responsible for lipid peroxidation, allowing it to successfully cure a variety of disorders. It has been demonstrated that caffeine increases the activity of antioxidant enzymes such SOD, GPx, GSH, and CAT, and also reduces the overproduction of ROS and RNS [175]. Furthermore, after delivery, striatal DA levels increased significantly, while inflammatory mediators dropped [176] (Table 26).

**Chrysin**

Chrysin is a flavonoid, a naturally produced polyphenolic substance. A wide range of foods, including fruits, honey, vegetables, mushrooms, plants, and blue *passionflower*s contain chrysin [180].

Chrysin’s anti-inflammatory and antioxidant abilities, and variety of pharmacological characteristics, have been researched for their neuroprotective effects. The bioavailability and amount that can be attained in rat cells and target tissues influence its adaptability of therapeutic efficacy. Increased DA levels are inversely linked with dopaminergic neuron mortality in both in vivo and in vitro research, supporting chrysin’s neuroprotective action. It also boosted DA levels in PD mousemodels and CGN cells treated with MPP+ and MPTP by inhibiting monoamine oxidase B [181].

Furthermore, in Barnes maze, rotational behavior models, chrysin greatly corrected motor and cognitive deficiencies in such animals [182]. Chrysin is thus a potential disease modulator that can also assist in the decrease of Parkinson’s disease symptoms (Table 27).

**Vanillin**

Vanillin, a phenolic aldehyde molecule, is a popular flavoring component that is found all over the world. It is employed by various plant species and is frequently used in food, drinks, pharmaceuticals, aromatherapy, and personal care items. Vanillin’s structure and key biological functions, such as vanillyl alcohol and vanillic acid, are well known [186]. It can simply pass the blood–brain barrier, preventing oxidative damage to the brain [187] (Table 28).

**Asiatic Acid**

As a neuroprotective drug, asiatic acid, a naturally occurring pentacyclic triterpenoid, has several pharmacological effects. Many biological roles of asiatic acid have been demonstrated, including antioxidative, anticarcinogenic, and neuroprotective characteristics. It protects against Parkinson’s disease by reducing mitochondrial oxidative stress [191] (Table 29).

**Thymoquinone**

Black cumin, as well as other Labiatae plants’ seeds, contains the pharmacologically active compound thymoquinone (TQ) [197]. Thymoquinone therapy reduced the mortality of primary dopaminergic neurons in a rotenone-induced Parkinson’s disease model, confirming the compound’s neuroprotective effect [198].

Thymoquinone dramatically increased the expression of neuroprotective proteins, decreased NF-B activation, and significantly reduced the expression of pro-inflammatory cytokines in LPS/IFN-activated BV-2 microglial cells. In addition, it reduces oxidative stress via the Nrf2/ARE signaling pathway, which delays dopaminergic neurodegeneration [199] (Table 30).

**Ferulic Acid**

Ferulic acid (FA) is a naturally occurring phenolic phytochemical that is present in a number of foods. Eating vegetables, fruits, and wholegrains has been shown to help prevent illnesses such as malignancy, obesity, coronary heart disease, and Parkinson’s disease [203]. It contains immunomodulatory, oxidative, chemotherapeutic, and therapeutic properties [204]. It also has a low toxicity level. Through decreasing lipid peroxidation and ROS production through the action of its phenolic hydroxyl group, ferulic acid is able to carry out these functions. It also suppresses the expression of enzymes that promote inflammation [203] (Table 31 and Table 32).

**Recent Clinical Trials**

Below are some of the phytoconstituents that have been used in various disorders that shown their effect in different clinical trials (Table 33).

**Phytochemical-Based Formulations**

Below are some phytochemical-based market formulations for the treatment of neurodegenerative disorders (Table 34).

**Novel Drug Delivery Approaches for Phytoconstituents**

**Figure 5:**
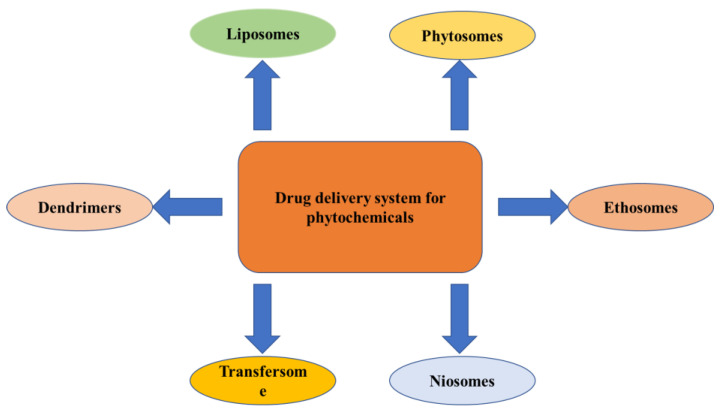
Novel drug delivery system for phytochemicals.

The novel techniques for the drug delivery of phytochemicals use liposomes, phytosomes, niosomes, transferosomes, ethosomes, etc., which are discussed below (Figure 5).

**Role of Nanotechnology in Various Neurodegenerative Disorders**

**Figure 6:**
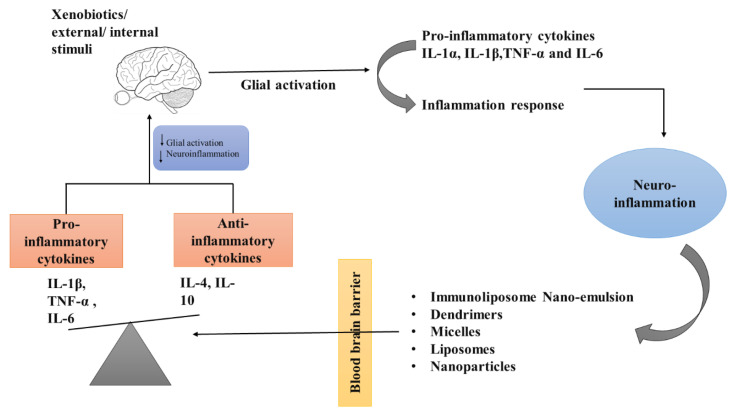
Role of nanoparticles in neurodegenerative disorders.

The therapy of neurodegenerative disorders such as AD, PD, stroke, epilepsy, and Huntington’s disease has been improved by the development of newer medication delivery strategies such as nanoparticles. The nanoparticles that are given have the ability to cross the blood–brain barrier and then target a particular cell or signaling pathway (Figure 6).

**Nanotechnology-Based Brain Targeting via Alteration of Blood–Brain Barrier**

The BBB is a selective permeability barrier that works as a local gateway for circulating antigens and foreign particles. This is the most complex gateway to be crossed and acts as a major challenge for the drug delivery vehicles to target the drug molecules to the brain. Emerging nanotechnologies, involve a number of fascinating concepts that have the potential to serve as the answer to this persistent challenge.

The advancement of nanotechnology through interdisciplinary collaboration will lead to new understandings of how neural circuits function, as well as the methods for the detection and treatment of brain disorders. A new drug delivery platform that makes it simple to transfer therapeutic compounds to the brain can be developed by utilizing the unique qualities of nanomaterials, such as their smaller size, biocompatibility, longer blood circulation, and lack of toxicity. Both specialized and general targeting strategies for brain locations are used in nanotechnology-mediated medication delivery systems. The formulation of nano-vehicles for the delivery of drugs, such as nanoparticles, liposomes, dendrimers, micelles, and carbon nanotubes, has received a lot of attention recently. These vehicles can deliver pharmaceuticals, peptides, proteins, vaccines, or nucleic acids [220].

**Alzheimer’s Disease**—Researchers have developed a wide variety of nano-formulations in the hopes that they will have a positive effect on AD patients. The studies that were conducted in vitro on the SHSY-5Y human neuroblastoma cell line showed that phospholipid-based PEG-stabilized nano-micelles inhibit Aβaggregation and reduce Aβ-induced neurotoxicity [230]. The phytochemical curcumin was shown to have the power to decrease Aβ oligomerization and cell proliferation in the in vitro study; however, when it was injected into mice, it showed poor bioavailability [231]. Curcumin was formulated by using nanoliposomes, which improved the bioavailability without compromising the drug’s ability to prevent Aβ aggregation [232].

**Parkinson’s Disease**—According to one study, L-Dopa and nerve-growth-factor-bound PBCA nanoparticles can cross the BBB and treat the primary signs and symptoms of Parkinson’s disease (PD) [233].

In addition, in another study, SA (Schisantherin A), which is used for the treatment of PD, was encapsulated in nanoparticles, which increasedits circulation in the blood stream and also its uptake in the brain. This showed that delivery with nanoparticles increases the efficacy of SA and makesit a potent compound for the treatment of PD [234].

**Huntington’s Disease**—Nitrendipine, a calcium channel blocker, has been demonstrated to significantly lower the incidence of dementia in HD patients over the duration of two years. However, this drug has permeability issues, due to its hydrophilic nature, and, as a result, it crosses the BBB very poorly. A study was conducted in which SLNs of nitrendipine were prepared, and a comparison of the uptake of nano-formulation and the bulk drug was carried out. According to the findings, the drug was taken in at a higher rate when it was encapsulated in SLNs [235].

**Epilepsy**—It has been discovered that solid lipid nanoparticles packed with carbamazepine and PLGA nanoparticles filled with β-carotene have the ability to have a greater anticonvulsant impact compared to polysorbate-80 and carbamazepine-packed, nanoemulged-covered nanoparticles [236].

In a rat model, subcutaneous injection of ethosuximide-encapsulated chitosan nano-capsules was found to reduce the spike-wave discharge. This finding was made by researchers using the rat as a model. Because they can provide a consistent release of the medication, these nanoparticles can be developed as depot drug delivery systems, enabling the long-term usage of antiepileptic drugs [237].

**Advantages of Various Novel Drug Delivery Methods**

There are various advantages of novel approaches for drug delivery using nanoparticles (Table 35).

## 2. Conclusions

Phytochemicals that are naturally derived and have been shown to be effective against a number of age-related neurodegenerative illnesses have been the focus of a growing number of studies in recent years. Because the treatment options for such illnesses with synthetic drugs in clinical studies have been demonstrated to be challenging, due to their toxicity and capacity to induce numerous different serious disorders, treatments with natural antioxidants, such as polyphenols via food or nutraceuticals, have become an excellent option to fight against the oxidative damage caused by free radicals to the neuronal cells. Although no major side effects of the phytochemicals that are now available have been observed clinically, there is a paucity of research in the field. In addition, Parkinson’s disease has been treated successfully with natural products; in particular, the phytochemicals obtained from naturally occurring foods have the ability to act as antioxidants and may serve as a dependable source of medication. However, naturally occurring lipophilic phytochemicals are able to enter the brain and cross the blood–brain barrier with relative ease. These phytochemicals offer enhanced bioavailability, a faster metabolism, and a better affinity to the receptors. Therefore, they can be taken on a consistent basis if one wishes to accelerate the process of neuronal function and reversal of the disease. In addition, when multiple neurotoxic models of Parkinson’s disease are combined, this showed that herbal therapies may be utilized in the development of novel Parkinson’s disease treatments. However, in the future, research should be conducted in real life to investigate the efficacy of plant extracts and the active components of those extracts in PD models. In addition, there is an ongoing demand for more in-depth explanations of the ingredients of herbal extracts, as well as the mechanisms through which they exert their effects. Furthermore, for the management of Parkinson’s disease (PD) by the use of natural products, methodological changes need to be implemented in order to guarantee high levels of reproducibility, increase the therapeutic effect, and reduce the likelihood of any potential harm.

## 3. Materials and Methods

A literature search was accomplished with electronic databases PubMed, Scopus, and Google Scholar, from which only English articles published until January 2023 were included. The succeeding search terms and Boolean connectors were used in the following search: “PD OR Parkinson’s disease OR neurodegenerative disorders OR neurodegenerative diseases OR NDD” AND “antioxidant OR antioxidant compounds OR natural antioxidant OR natural antioxidants”. These terms were searched in the abstract, title, or keywords. From the searched results, examples of natural antioxidant compounds had been studied, which were effective in neurodegenerative disorders, and the most were selected. The inclusion criteria for the articles were as follows: (i) research that uses natural antioxidants in the management of neurodegenerative disorders, including PD; (ii) their mechanism of action; (iii) invitrostudies performed; (iv) phytochemical-based formulations; (v) research that includes the novel approaches for the drug delivery of phytochemicals; (vi) recent clinical trials on phytochemicals; and (vii) the chemical structure of phytochemical used in PD. Studies without protein or mechanism analysis were excluded. However, in this review, we have tried to include phytochemicals that can be used to manage Parkinson’s disease.

## Figures and Tables

**Table 1:** Side effects of synthetic drugs.

Synthetic Drug	Toxicity	References
Levodopa	Motor complications, dyskinesias, motor fluctuations	[19]
Dopamine Agonists	Hallucinations, impulse control disorders, excessive daytime sleepiness	[20]
MAO-B Inhibitors	Potential hypertensive crises, interactions with medications and certain foods	[21]
Anticholinergic Drugs	Cognitive decline, memory impairment, dry mouth, urinary retention	[22]
COMT Inhibitors	Hepatotoxicity, liver dysfunction	[23]

**Table 3:** Some other potent activities of Ascorbic acid.

Compound Name	Disease	Impact	Ref.
Ascorbic acid	Heart disorder	Prevents stroke	[56]
	Respiratory disorder	Prevents asthma	[57]
	Cataract	Prevents the occurrence of cataracts	[58]

**Table 4:** Some other potent activities of Flavonoids.

Compound Name	Disease	Impact	Ref.
Flavonoids	Inflammation	Prevents diseases such as sclerosis, atherosclerosis, psoriasis, and ileitis/rheumatoid arthritis	[62]
	Bacterial infection	Inactivates cell envelope transport proteins	[63]
	Cancer	Treats cancer by interfering in the pathway of signal transduction	[64]

**Table 5:** Some other potent activities of Green tea polyphenols.

Compound Name	Disease	Impact	Ref.
Green Tea Polyphenols	Cancer	Defends against malignancies of the skin, breasts, colon, liver, lungs, esophagus, duodenum, and pancreas that are brought on by chemical carcinogens	[67]
	Cardiovascular diseases	Reduces the risk of coronary artery disease	[68]
	Neurodegenerative disorders	Decreases cognitive disorders	[69]

**Table 6:** Some other potent activities of Isoflavones.

Compound Name	Disease	Impact	Ref.
Isoflavones	Cancer	Shows protective effect against postmenopausal breast cancer	[72]
	Osteoporosis	Recovers bone mineral density	[73]
	Coronary heart disease	Reduces LDL levels	[74]

**Table 7:** Some other potent activities of Nicotine.

Compound Name	Disease	Impact	Ref.
Nicotine	Neurodegenerative disorders	Prevents motor functions	[77]
	Alzheimer’s disease	Raises cognitive functions	[78]
	Parkinson’s disease	Improves Parkinson’s disease symptoms	[79]

**Table 8:** Some other potent activities of Carotenoids.

Compound Name	Disease	Impact	Ref.
Carotenoids	Cancer	Reduces the cancer risk	[82]
	Heart disorders	Shows a reduction in myocardial infarction	[83]
	Photosensitivity	Prevents pigmentation, telangiectasia, dryness, and skin inelasticity	[84]

**Table 9:** Some other potent activities of Resveratrol.

Compound Name	Disease	Impact	Ref.
Resveratrol	Cardiovascular disorders	Shows pleiotropic effects	[88]
	Renal disorders	Prevents kidney diseases	[89]
	Stroke	Prevents stroke	[90]

**Table 10:** Some other potent activities of Tannins.

Compound Name	Disease	Impact	Ref.
Tannins	Diabetes	Enhances glucose uptake and inhibits adipogenesis	[93]
	Bacterial infection	Showsan antimicrobial impact on a range of Gram-positive andGram-negative bacteria	[94]
	Neurodegenerative and neuropsychiatric diseases	Shows anti-inflammatory, antioxidative, and anti-cholinesteraseeffects	[95]

**Table 11:** Some other potent activities of Curcumin polyphenol.

Compound Name	Disease	Impact	Ref.
Curcumin Polyphenol	Chronic fatigue syndrome	Significantly decreases immobility time and hyperalgesia	[99]
	Parkinson’s Disease	Shows antioxidant, anti-inflammatory, and anti-cancer effects	[100]
	Cognitive impairment	In the rat hippocampus, curcumin, at low or high dosages, could prevent neurotoxicity	[101]

**Table 12:** Some other potent activities of Ginkgo.

Compound Name	Disease	Impact	Ref.
Ginkgo biloba	Cardiovascular disease	Decreases platelet aggregation and thrombin formation	[104]
	Retinal diseases	Shows an antioxidant effect for the treatment of retinal diseases associated with neurodegeneration	[105]
	Neurological disorders	Effectively treatsa variety of human illnesses, such as those impacting brain function	[106]

**Table 13:** Some other potent activities of Pomegranate.

Compound Name	Disease	Impact	Ref.
Pomegranate	Cancer	It demonstrates anti-metastatic, anti-invasive, and antiproliferative effects on cancer	[109]
	Type 2 diabetes	Managestype 2 diabetesby reducingoxidative stress and lipid peroxidation	[110]
	Obesity	Increases PPAR-a, and decreases oxidative stress and lipid peroxidation	[111]

**Table 14:** Some other potent activities of Baicalein.

Compound Name	Disease	Impact	Ref.
Baicalein	Parkinson’s Disease	Decreases excitotoxicity, promotes neurogenesis and cell differentiation, prevents the accumulation of amyloid proteins that are associated with illness, and has antiapoptotic actions	[114]
	Alzheimer’s	The total times of rotating on the rotarod, T-maze, and cage activity, were all significantly reduced by baicalein. In addition, histopathology proves the effectiveness of baicalein against Alzheimer’s	[115]
	Inflammatory disorders	Up-regulation of the relevant cytokines and chemokines from the target sites will improve antioxidant status and reduce oxidative stress in damaged tissues	[114]

**Table 15:** Some other potent activities of *Peganum harmara*.

Compound Name	Disease	Impact	Ref.
*Peganum harmara*	Cancer disorder	Exhibits anti-inflammatory and anti-cancer capacities	[117]
	Alzheimer’s Disease	Enhances endogenous antioxidant enzymes, decreasesthe ACTH effect in the brain, and inhibits lipid peroxidation to improve neurodegeneration	[118]
	Arthritis	Prostaglandin-E2 and TNF levels in the serum of polyarthritic rats were significantly improved by the plant extract	[119]

**Table 16:** Some other potent activities of *Carthamus tinctorius* L.

Compound Name	Disease	Impact	Ref.
*Carthamus tinctorius* L. (Safflower)	Diabetes	The plant extract stimulates the secretion of insulin from the beta cells	[121]
	Foliar fungal	Oxidative stress resistance was induced to fight the foliar fungal disease complex	[122]
	Parkinson’sdisease	Mogamiflower benibana’s extract exhibits neuroprotective and free-radical-scavenging properties	[123]

**Table 17:** Some other potent activities of *Pueraria lobata*.

Compound Name	Disease	Impact	Ref.
*Pueraria lobata*	Diabetes	Increased expression of glucose transporter type 4 enhances glucose absorption in L6 cells and boosts glucose utilization activity (GLUT4)	[126]
	Liver diseases	Controls lipid metabolism, inflammatory response, and oxidative stress	[127]
	Inflammation	Lupenone and lupeol efficiently reduced the production of NO and the expression of iNOS and/or COX-2 in LPS-stimulated RAW 264.7 cells	[128]

**Table 18:** Some other potent activities of Ginseng.

Compound Name	Disease	Impact	Ref.
Ginseng	Cardiovascular diseases	Ginseng has the capacity to manage cardiac conditions by controlling vasomotor function, enhancing lipid profiles, modulating ion channels and signal transduction, adjusting blood pressure, and reducing platelet adhesion, according to in vitro and in vivo investigations	[131]
	Liver disease	Ginseng suppresses the signals of nuclear factor-kappa-B (NF-kB) and mitogen-activated protein kinase (MAPK)	[132]
	Alzheimer’s	When compared to the control group, ginseng-treated groups showed improvement from the baseline MMSE	[133]

**Table 19:** Some other potent activities of *Passionflower*.

Compound Name	Disease	Impact	Ref.
*Passionflower*	Anxiety	A significant reductionwas foundafter one monthin auditory omission errors in the *passionflower* group	[136]
	Parkinson’s disease	Due to anti-Parkinson’s activity, there is a significant decrease in the tacrine-induced jaw and haloperidol-induced catalepsy movements	[135]
	Neuropsychiatric disorders	The passionflower plant may be able to treat problems with neuropsychiatric roots	[137]

**Table 20:** Some other potent activities of St. John’s Wort.

Compound Name	Disease	Impact	Ref.
St. John’s Wort (SJW)	Somatoform disorders	St. John Wort extract is effective and safe for the treatment of somatoform disorder	[140]
	Depression	For treating mild to moderately severe depressive disorders, extracts are more effective than a placebo	[141]
	Alzheimer’s	A highly elevated export activity in the blood–brain barrier results in a reduction in soluble A42 species	[142]

**Table 21:** Some other potent activities of *Bacopa monnieri*.

Compound Name	Disease	Impact	Ref.
*Bacopa monnieri*	Alzheimer’s	The clinical investigation demonstrates that taking the extract for six months improves several elements of cognitive function in elderly Alzheimer’s patients	[144]
	Parkinson’s disease	Decreases alpha synuclein aggregation, restores the lipid content in nematodes, and prevents dopaminergic neurodegeneration	[145]
	Diabetes	Rat models treated with an ethanolic extract of *B. monnieri* exhibited significant improvements in body weight and glycemic index compared to the control rats	[146,147]

**Table 22:** Some other potent activities of *Mushrooms*.

Compound Name	Disease	Impact	Ref.
*Mushrooms*	Cancer	Inhibiting cancer cell metastasis and growth	[152]
	Skin disorder	Effective in the treatment of skin disorders such as vitiligo	[153]
	Inflammation	In activated macrophages, *Mushroom* extracts reduced iNOS expression and NO generation	[154]

**Table 23:** Some other potent activities of *Sea cucumbers*.

Compound Name	Disease	Impact	Ref.
*Sea Cucumbers*	Viral	Antiviral potential	[157]
	Hypertension	Inhibit ACE and regulate blood pressure	[158]
	Hyperlipidaemia	Reduce high blood cholesterol levels	[159]

**Table 24:** Some other potent activities of Oleuropein.

Compound Name	Disease	Impact	Ref.
Oleuropein	Hypertension	Lowers blood pressure	[163]
	Cancer	Inhibits inflammation and initiates apoptosis	[164]
	Oleuropein	Cardioprotective against ischemic—reperfusion injury	[165]

**Table 25:** Some other potent activities of Theaflavin.

Compound Name	Disease	Impact	Ref.
Theaflavin	Cancer	Promotes programmed cell death	[169]
	Obesity	Reduces function of adipocytes	[170]
	Diabetes	Reduces glucose levels	[171,172]

**Table 26:** Some other potent activities of Caffeic acid.

Compound Name	Disease	Impact	Ref.
Caffeic Acid	Inflammation	Inhibits inflammatory mediators	[177]
	Cancer	Decreases metastases in tumor cells	[178]
	Photoaging	Increased MMP-1 and MMP-9 expression in human skin fibroblasts is prevented by blocking UVB exposure	[179]

**Table 27:** Some other potent activities of Chrysin.

Compound Name	Disease	Impact	Ref.
Chrysin	Cancer	Inhibits cancer cell growth	[183]
	Diabetes	Prevents poor glucose tolerance and insulin signaling molecules	[184]
	Inflammation	Reduces the level of interleukin-1β (IL-1β), TNF-α, and interferon gamma (IFN-γ), IL-12	[185]

**Table 28:** Some other potent activities of Vanillin.

Compound Name	Disease	Impact	Ref.
Vanillin	Alzheimer’s disease	Inhibits Aβ amyloid aggregation	[188]
	PD	Provides neuroprotection and anti-inflammatory action	[189]
	Huntington’s Disease	Reduces weight loss and impaired motor coordination caused by 3-NPA	[190]

**Table 29:** Some other potent activities of Asiatic acid.

Compound Name	Disease	Impact	Ref.
Asiatic Acid	Wounds	Helps to heal wounds and increases the speed of nerve regeneration	[192]
	Inflammation	Displays an anti-inflammatory effect	[193,194]
	Diabetes	Advanced glycation end products’ emergence is retarded	[195,196]

**Table 30:** Some other potent activities of Thymoquinone.

Compound Name	Disease	Impact	Ref.
Thymoquinone	Inflammation	Reduces inflammation in brain cells	[200]
	AD	Improves learning and memory	[201]
	Ischemia	Provides protection against transient forebrain ischemia	[202]

**Table 31:** Some other potent activities of Ferulic acid.

Compound Name	Disease	Impact	Ref.
Ferulic Acid	Cardiovascular disorders	Reduces hypercholesterolemia and hyperglycemia	[205]
	Viral diseases	Produces antibodies against the influenza virus	[206]
	Diabetes	Exhibits antidiabetic action	[207,208]

**Table 32:** Role of different natural antioxidants with their chemical structure and origin.

Chemical Name	Chemical Structure	Plant Extract	Mechanism of Action	Ref.
**Baicalein**	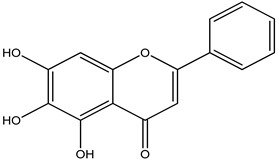	Roots of *Scutellaria baicalensis* and *Scutellarin lateriflora*	Reducesoxidative stress, inhibiting aggregation of amyloid proteins	[112]
**Curcumin**	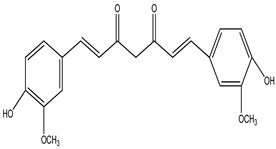	*Curcuma longa*	They have anti-inflammatory, chemotherapeutic, antioxidant, antiproliferative, wound-healing, and antiparasitic properties	[209,210,211]
**Resveratrol**	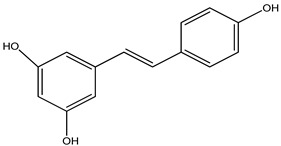	*Polygonum cuspidatum*	Improves motor impairments and reduces oxidative stress	[85]
**Ginkgolides**	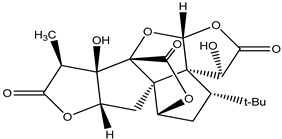	*Ginkgo biloba*	Is an ntioxidant and decreases oxidative stress	[112]
**L-DOPA**	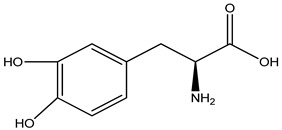	*Mucuna pruriens*	Both the brain and peripheral areas convert it to dopamine	[212]
**Nicotine**	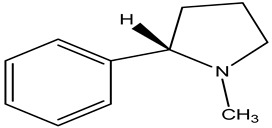	*Nicotiana tabacum*	Nicotine reduces SIRT6 levels in brain tissue and neural cells	[75]
**St. John’s Wort**	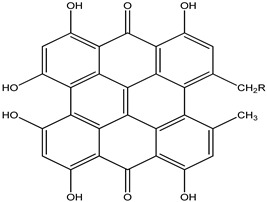	*Hypericum perforatum*	Shows antioxidant and antidepressant effects	[138]
**Vitamin C**	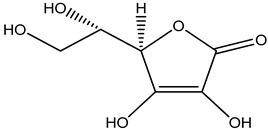	*Citrus limon*	Antioxidant	[213]
**Vitamin E**	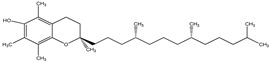	*Prunus amygdalus*	Antioxidant	[214]
**Isoflavone**	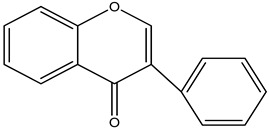	Chamomile	An antioxidant, preventing mitochondrial oxidative stress	[215]
**Punicalin**	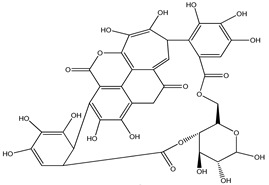	*Punica granatum*	Shows antioxidative, anti-inflammatory, and antiapoptotic activity	[108]
**Oleuropein**	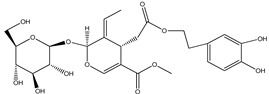	Olive oil	Inhibits the buildup of ROS	[161,162]
**Theaflavin**	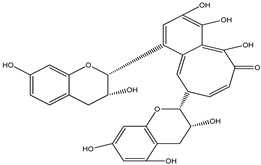	Black tea	Removes excess free radical production and metal chelation	[167,216]
**Caffeic acid**	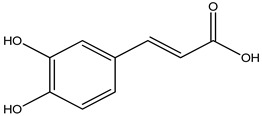	Coffee	Eliminates excess ROS/RNS production	[217]
**Ferulic acid**	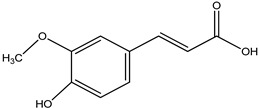	Peanuts	Inhibitslipid peroxidation and ROS production	[203,218]

**Table 33:** Recent clinical trials.

Compounds	Uses	Id	Trial Phase	Ref.
**Pinocembrin**	Ischemic stroke	NCT02059785	II	[219]
**EGCG**	Ischemic stroke, Alzheimer’s disease, and Huntington’s disease	NCT00951834, NCT01357681	II	[220]
**Quercetin**	Reduces brain injuries through a variety of methods, such as antiapoptosis, autophagy advancement, and significant metabolic regulation	NCT01376011, NCT01376011	I	[221]
**Curcumin**	Alzheimer’s, cerebral infarction, and brain edema	NCT00164749, NCT01001637	II	[222]
**Cannabidiol**	Motor/tremor improvement	NCT03582137, NCT02818777	II	[223]
**Borneol**	Can bring back consciousness when the brain has been injured	NCT03495206	I	[224]
**Ginsenoside Rd**	Helpful in neuroprotection	NCT00591084, NCT00815763	III	[225]
**Vinpocetine**	Effect on preventing strokes caused by ischemia	NCT02878772	III	[226]

**Table 34:** Marketed formulations.

Formulation	Composition	Function	Dose	Ref
**Mentat (BR-16A)**	Brahmi, Ashwagandha, Mandookaparni, Shankapushpi, Jatamansi, Vach, Tagar, Badam, Salap, Lavang, Pearl, Malkangni, Sonth	Memory enhancement, cognitive deformities	100 mg/kg/day	[227]
**Trasina**	*Withania somnifera*, *Ocimum sanctum*, *Eclipta alba*, *Tinospora cordifolia*, *Picrorrhiza kurroa*, Shilajit	Enhance cholinergic markers and memory	200 and 500 mg/kg	[227]
**Memorin**	Mandookparni, Shankhpushpi, Jatamansi, Yashtimadhu, *Smruti sagar*	Boost your memory	200 mg/day/kg	[227]
**Bramhi Ghrita**	*Bacopa monneri*, *Evolvulus alsinoids*, *Acorus calamus*, *Saussurea lappa*, Cow’s ghee	Improve learning ability	30, 50, and 100 mg/kg	[228]
**Abana**	*Terminalia arjuna*, *Withania somnifera*, *Nepeta hindostana*, Dashamoola, *Tinospora cordifolia*, *Phyllanthus emblica*, *Terminalia chebula*, *Eclipta alba*, *Glycyrrhiza glabra*, *Asparagus racemosus*, *Boerhaavia diffusa*, Shilajeet, *Centella asiatica*, *Convolvulus pluricaulis*, *Ocimum sanctum*, *Nardostachys jatamansi*, *Piper longum*, *Carum copticum*, *Zingiber officinale*, *Shankh bhasma*, Makardhwaj, *Cyperus rotundus*, *Acorus calamus*, *Embelia ribes*, *Syzygium aromaticum*, *Celastrus paniculatus*, *Santalum album*, *Elettaria cardamomum*, *Foeniculum vulgare*, *Rosa damascene*, *Cinnamomum cassia*, Jaharmohra, *Abhrak bhasma*, *Akik pishti*, *Yeshab pishti*, *Yakut pishti*, *Praval pishti*, *Crocus sativus*	Memory enhancement	200 mg/kg	[229]

**Table 35:** Advantages of various novel drug delivery methods.

Drug Delivery Method	Advantages	References
**Liposomes**	Capable of transportingboth hydrophillic and hydrophobicdrugs. Easily transport cytotoxic materials. Easily carry sustained and controlled release medicaments.	[229]
**Phytosomes**	Enhance the bioavailability of hydrophilic phytoconstituents, enhance the drug entrapment power, and also enhance absorption. Utilizable in the cosmetics industry.	[238]
**Niosomes**	Cost less than liposomes. Easy to transport	[239]
**Proniosomes**	Less likely to degrade during the process of storing and sterilizing. Simple in terms of transfer and distribution.	[239]
**Ethosomes**	Increase absorption through the skin. Deliver large quantities of drugs.	[240]

## Data Availability

Not applicable.
